# HER-2 Expression in Brain Metastases from Colorectal Cancer and Corresponding Primary Tumors: A Case Cohort Series

**DOI:** 10.3390/ijms14022370

**Published:** 2013-01-24

**Authors:** Giuseppe Aprile, Giovanna De Maglio, Jessica Menis, Mariaelena Casagrande, Francesco Tuniz, Federica Edith Pisa, Caterina Fontanella, Miran Skrap, Carlo Alberto Beltrami, Gianpiero Fasola, Stefano Pizzolitto

**Affiliations:** 1 Department of Oncology, University and General Hospital, 33100 Udine, Italy; E-Mails: jessica.menis@libero.it (M.J.); mericasagrande@libero.it (C.M.); c.fontanella@yahoo.it (F.C.); fasola.gianpiero@aoud.sanita.fvg.it (F.G.); 2 Department of Pathology, University and General Hospital, 33100 Udine, Italy; E-Mails: demaglio.giovanna@aoud.sanita.fvg.it (D.M.G.); pizzolitto.stefano@aoud.sanita.fvg.it (P.S.); 3 Department of Neurosurgery, University and General Hospital, 33100 Udine, Italy; E-Mails: tuniz.francesco@gmail.com (T.F.); skrap@aoud.sanita.fvg.it (S.M.); 4 Institute of Hygiene and Epidemiology, University and General Hospital, 33100 Udine, Italy; E-Mail: federica.pisa@uniud.it; 5 Institute of Pathology, University and General Hospital, 33100 Udine, Italy; E-Mail: beltrami@uniud.it

**Keywords:** colorectal cancer, brain metastases, neurosurgery, HER-2, survival

## Abstract

Brain metastases (BM) from colorectal cancer (CRC) are a rare but increasing event. Surgical resection of oligometastatic disease, including BM, may produce a survival benefit in selected patients. Previous studies described the HER-2 expression patterns in CRC patients, but its prognostic role still remains controversial. Information on the HER-2 expression in BM from CRC is currently lacking. Among the over 500 patients treated at our Department of Neurosurgery in the last 13 years (1999–2012), we identified a cohort of 50 consecutive CRC patients resected for BM. Clinical data were retrospectively reviewed using electronic hospital charts and surgical notes. Formalin-fixed, paraffin-embedded tissue samples were retrieved and histologically reviewed. HER-2 status was assessed on 4-μm sections by HerceptTest™, and scored by two pathologists according to gastric cancer HER-2 status guidelines. In score 2+ cases HER-2 gene copy number was analyzed by FISH, performed using the PathVysion HER-2 DNA Probe Kit. Median age at time of BM resection was 65 years (35–82); most patients were males (60%) with a good performance status. The majority of the BM were single (74%) and sited in the supratentorial area (64%); 2–4 lesions were diagnosed in 9 patients (18%), and >4 in 3 patients (6%). The rate of HER-2 positivity (defined as IHC score 3+ or IHC score 2+ and FISH gene amplification) was 8.1% for the primary CRC tumors and 12% for their corresponding BM. The concordance rate between primary tumors and matched BM was 89%. Median overall survival after neurosurgery was 6.5 months for HER-2 IHC score 0 *vs.* 4.6 months for HER-2 IHC score 1+/2+/3+; the difference was statistically significant (*p* = 0.01, Log-rank test). HER-2 positivity of our case cohort was low but comparable to literature. Concordance rate of HER-2 expression between BM and corresponding primary tumors is high and similar to those reported for breast and gastric cancers. Our data suggest a potential negative prognostic value of HER-2 expression in brain lesions from CRC.

## 1. Background

Brain metastases (BM) from colorectal cancer (CRC) are a rare but increasing event, reported to occur in less than 5% of patients at the time of diagnosis and in up to 10% within the course of treatment [1]. Nevertheless, their incidence is expected to rise as a consequence of the improved survival reported for patients with metastatic CRC, that reflects the increased number of available medical treatments [2] and the significant progress in surgical techniques [3]. The median overall survival (OS) of advanced CRC patients varies according to the chance of radically resection of all metastatic lesions. Currently, up to 25% of suitable patients may safely undergo surgical resection with curative intent [4]. Indeed, the radical resection of hepatic [5] or pulmonary metastases [6] can provide long-term survival in carefully selected patients, yielding to a 5-year survival rate of 36%–58% and 27%–41%, respectively [7]. The median OS of CRC patients with BM is about 4 months, longer for those who receive surgical treatment, and shorter for those treated with chemotherapy or supportive care alone [1]. As previously reported [3,8–10], we also showed that resection of isolated or symptomatic BM, along with whole-brain radiation, may prolong survival [11]. Other favorable prognostic factors include younger age (<65 years), good performance status (PS), lower Recursive Partitioning Analysis class, the presence of a solitary brain lesion, and the absence of uncontrolled systemic disease [1].

HER-2 (ErbB-2, c-erbB2 or Her2/neu) is a proto-oncogene located on the chromosome 17 at q21. It encodes a 185-kDa trans-membrane tyrosine kinase receptor, a member of the HER-family that also includes HER-1 (Epidermal Growth Factor Receptor-EGFR, or ErbB1), HER-3 (ErbB3) and HER-4 (ErbB4). HER-2 gene amplification and protein overexpression are involved in the pathogenesis and progression of several human cancers [12] and represent, in most of them, a poor prognostic factor [13]. Also in gastrointestinal tumors, and particularly in esophagogastric junction and gastric adenocarcinomas, evidence exists that c-erbB2 is overexpressed (in about 25% and 15%, respectively). HER-2 positivity is a poor prognostic factor in esophageal cancers [14] while its prognostic value is less clear in gastric carcinomas [15–19]. In spite of some studies that failed to find an association with prognosis [20,21], increasing evidence suggests a potential role of HER-2 as a negative prognostic factor in gastric cancer [16–19].

Various members of the EGFR family and their ligands have been shown to mediate breast cancer metastatic spread to the brain in a multistep process, including blood–brain barrier infiltration, extravasation, and brain colonization [22,23]. HER-2 amplification or strong overexpression correlate with increased risk for BM, and HER-2 positive breast cancer patients develop BM more frequently and rapidly than those with HER-2 negative tumors [24,25], especially if ER and PgR negative [26,27]. Even in esophageal cancer HER-2 overexpression seems to be associated with an increased risk for BM [28].

HER-2 amplification occurs in a very small percentage (around 3%) of genetically unselected CRC [29–31] thus limiting the usefulness of HER-2 inhibition in the overall population [32–34]. However, preclinical studies [35,36] and clinical trials [37] showed that its incidence increases in KRAS wild-type CRC patients resistant to EGFR-inhibitors.

Barbara and colleagues found 4.5% HER-2 gene amplification in a cohort of 266 metastatic CRC patients treated with cetuximab or panitumumab; HER-2 gene amplification was significantly related to therapy resistance (*p* < 0.0001), shorter progression-free survival (PFS, *p* = 0.0025), and reduced OS (*p* = 0.062) [37]. However, the prognostic role of HER-2 expression remains unclear. According to a German study [38], patients with locally advanced rectal cancer with high HER-2 overexpression had significantly better long-term survival when compared to those with low expression (73.2% *vs.* 60.1%; *p* = 0.0277).

Information on the HER-2 expression in BM from CRC is currently lacking. In this study, we described the expression of HER-2 in cerebral metastases, gathering clinical and pathological data from 50 CRC patients who underwent neurosurgery in the past 13 years (1999–2012).

## 2. Results

All 50 CRC patients, neurosurgically resected for their BM, were considered eligible and included in our analysis. Median age at time of BM resection was 65 years (35–82); most patients were males (*n* = 30, 60%) and presented with a single brain lesion (*n* = 37, 74%). Patients were followed for a median of 48 months. Complete demographics and patients’ clinical characteristics are summarized in Table 1. At diagnosis, most patients presented with locally advanced or advanced disease (*n* = 19, 38% stage III; *n* = 19, 38% stage IV); only 1 patient was stage I (2%), and 3 patients were stage II (6%). At the time of BM diagnosis, all patients had systemic extra-cranial disease, the most frequent locations being liver, lungs, lymph-nodes, and peritoneum. Seventy-four percent of the primary tumors were resected: in 19 patients the tumor was located in the colon, with 17 patients it was in the rectum, while a clear distinction was not possible in 14 cases (Table 1). Only 3 patients with locally advanced disease received neoadjuvant radiotherapy before rectal surgery. Indeed, preoperative chemoradiation was not considered a clear standard until recent years, and some patients with rectal cancer did not receive any preoperative treatment because of the evidence of synchronous metastatic lesions. Adjuvant chemotherapy was administered to 40% of the enrolled patients and at least 58% of the patients received any palliative chemotherapy; the median number of palliative chemotherapy lines was one (Table 2). 5-Fluorouracil, the key therapeutic drug in both settings (60% of patients received it), was combined with oxaliplatin in 16 cases (32%) or irinotecan in another 16 patients (32%) (Table 2). Cetuximab was the only biologic agent used in combination with chemotherapy in four cases (Table 2). Only 10% of the BM were present at first diagnosis of advanced disease. The majority were single (74%) and sited in the supratentorial area (64%); two to four lesions were discovered in nine patients (18%) and multiple (>4) in three patients (6%) (Table 1). After neurosurgery, 21 out of 50 patients received postoperative whole brain radiotherapy (WBRT, 10 Gy in 5 fractions); five patients received gamma-knife, soon after the neurosurgical treatment, or at the time of cerebral progression (Table 3).

**Figure 1:**
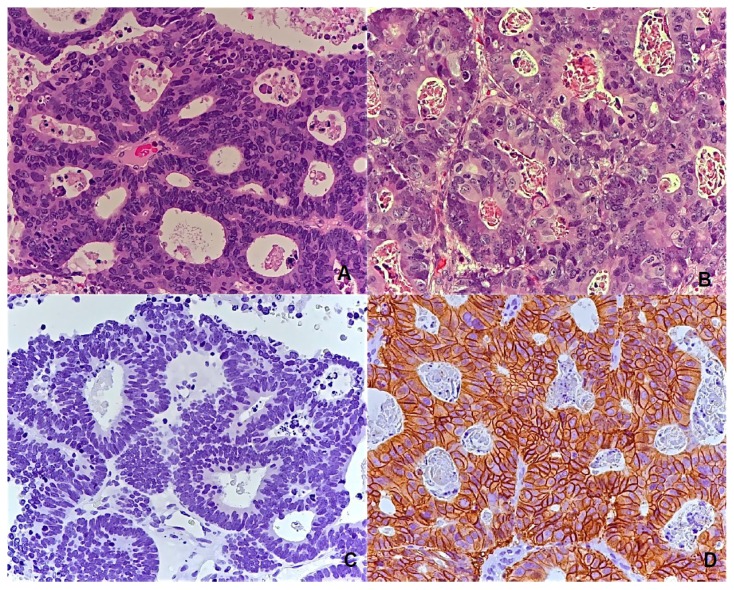
Hematoxylin-Eosin colorectal specimens (**A**) and paired immunohistochemical HER-2 staining scored 0 (**C**); Hematoxylin-Eosin colorectal specimens (**B**) and paired immunohistochemical (HER-2 staining scored 3+) (**D**) Magnification 20X.

IHC HER-2 expression pattern of the BM is described in Table 4: 36 cases score 0 (72%), eight cases score 1+ (16%), two cases score 2+ (4%) and both had FISH gene amplification, and four cases score 3+ (8%). Figure 1 shows the comparison of the HE and IHC slides for HER-2 score 0 and 3+.

HER-2 status on the primary tumor was negative in 34 cases (31 scored 0, three scored 1+), and positive in three cases (two scored 2+ with FISH amplification, one scored 3+); data was not available in 13 cases, in 10 of which the primary tumor had never been removed (Table 4). Comparison of HER-2 status was therefore possible in 37 cases (74% of the patients); concordance (*i.e.*, both negative or both positive) between primary tumor and corresponding BM was demonstrated in 89% of the matched samples. Table 5 depicts the concordance distribution: 31 out of 37 cases (83.7%) were HER-2 negative both on primary tumors and BM, three (8.1%) were HER-2 negative at diagnosis and became positive on brain lesions, and one (2.7%) was HER-2 positive and became HER-2 negative. Two cases (5.3%) were positive both on primary colorectal cancer and corresponding BM.

**Figure 2:**
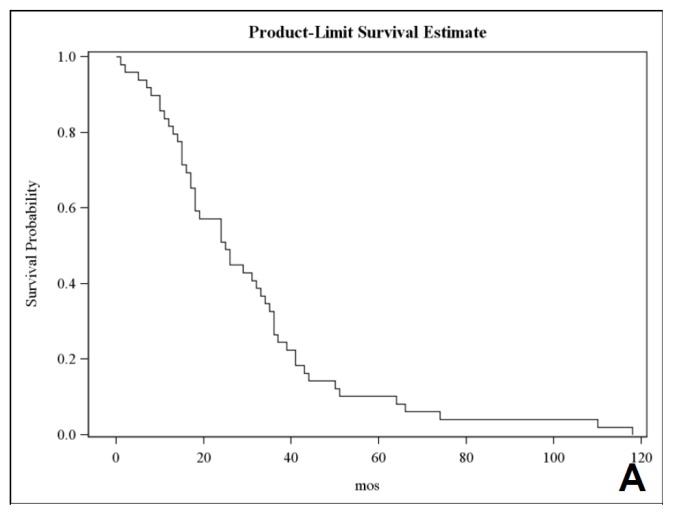

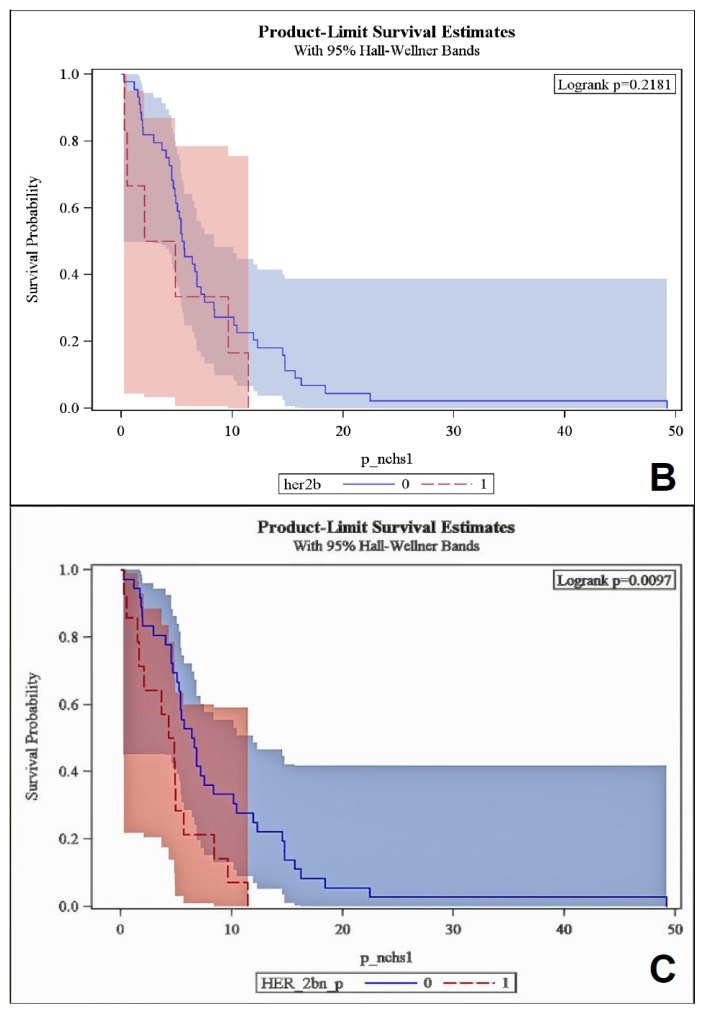
Overall survival of the whole study population (**A**); Survival postneurosurgery; (**B**) BM HER-2 0 (score 0/1+) *vs.* 1 (score 2+/3+); Survival postneurosurgery; (**C**) BM HER-2 0 (score 0) *vs.* 1 (score 1+/2+/3+).

In general, the median OS of the cohort was 25.5 months (95% CI 18–33 months) (Figure 2A). Focusing on the prognostic value, we firstly compared the outcome of HER-2 negative tumors (IHC score 0 or 1+) *vs.* HER-2 positive tumors (IHC score 3+ or 2+ with gene amplification at FISH): median postNCH-OS was 5.5 months *vs.* 3.4 months respectively (*p* = 0.18, Figure 2B); 6-month postNCH-OS rate was 45.5% *vs.* 33.3%, respectively.

Thereafter, considering the small number of HER-2 positive BM (*n* = 6), we arbitrarily decided to compare HER-2 score 0 tumors to HER-2 score 1+/2+/3+ tumors with a speculative purpose. Median DFS was 8.2 months for HER-2 IHC score 0 *vs.* 5.3 months for the others (*p* = 0.61). The rates of disease control at six or 12-months were higher for IHC negative tumors (52.8% *vs.* 42.8% and 36% *vs.* 28%, respectively). Median BPFS was 21.9 months for HER-2 0 *vs.* 11.8 months for HER-2 score 1+/2+/3+ (*p* = 0.34). Accordingly, median ΔBPFS-DFS was longer for tumors without HER-2 expression (10 *vs.* 4 months), but once more, not statistically different. Median OS was 30 months for HER-2 score 0, and 16 months for HER-2 1+/2+/3+ (*p* = 0.078). At 12 months 86.1% of HER-2 0 *vs.* 71.4% of HER-2 1+/2+/3+ were alive. Importantly, median postNCH-OS was 6.5 months for HER-2 score 0 *vs.* 4.6 months for HER-2 1+/2+/3+; the difference was statistically significant (*p* = 0.01) (Figure 2C). At six months from brain surgery, 52.8% of HER-2 score 0 were alive *vs*. 21.4% of HER-2 1+/2+/3+. The trend was confirmed at 12 months. One year after neurosurgery, 25% of CRC patients without HER-2 expression were still alive, while no patients with HER-2 expression survived.

## 3. Discussion

Among the reasons for the limited activity of current systemic therapies in the treatment of patients with BM, it has to be considered that the blood-brain barrier may prevent antitumor agents from penetrating the brain in sufficiently high concentrations to produce substantial effects. As a consequence, the choice for optimal treatment strategy and the study of predictive factors have become critical issues.

HER-2 is a well-known prognostic and predictive factor, and patients with HER-2 positive breast tumors have an increased risk for developing BM [39,40]. It also predicts survival in gastric cancer but here its prognostic role is still uncertain [16,18,21,41].

Our analyses aimed to describe the expression pattern of HER-2 in CRC BM, to compare HER-2 overexpression in BM and in corresponding primary tumors, and to explore its possible prognostic value. Less than 10% of our patients were treated with EGFR-inhibitors and none of them received antiangiogenetics. Yet, median OS was 25.5 months, similar to that reported in modern prospective randomized trials [7]. This data suggests how a favourable biological selection may overcome the limit of what would now be considered a suboptimal treatment.

The rate of HER-2 positivity in genetically unselected CRC is lower than those reported for breast or gastric cancers [13,41,42], accounting for a few percent of all cases. However, it may become higher in specific CRC populations. In our case series, the overall HER-2 positivity of BM was 12%. With regards to primary tumors, the rate of HER-2 positivity was 8%, analogous to the 6% and 7% recently reported by other investigators [43,44].

Concordance rate of HER-2 positivity between primary tumors and matched metastases exceeds 90% in breast cancers [45] and in gastric carcinomas [17,31]. In this latter disease, the heterogeneity of HER-2 amplification may be the reason for discordant HER-2 status of primary and matched metastases [42]. Our study was the first to analyze the HER-2 expression pattern in BM from CRC and to evaluate the concordance to their paired primary tumors. In 37 available cases, a 89% concordance rate was found. Likewise, a 97% concordance rate for HER-2 expression between primary breast cancer and corresponding cerebral metastases was reported [46]. Only one patient had a HER-2 positive CRC primary and a HER-2 negative matched secondary cerebral lesion. This patient, developed a single brain lesion 19 months after the diagnosis of advanced disease. His survival was very short (eight days) due to post-neurosurgical complications. Cases of HER-2 conversion from positive primary to negative metastases were reported elsewhere [38]. A similarly high HER-2 concordance rate was identified for hepatic metastases and their corresponding CRC primaries [47].

The prognostic role of HER-2 in CRC is unclear, and conflicting results have been reported. While some studies suggested a worse prognosis for HER-2 positive CRC patients [48–50], other reports did not show any difference in overall survival [45,51]. Previous studies found a significant correlation between stage and HER-2 positivity, HER-2 being more common in advanced stage [48,49]. Also, a significant correlation between HER-2 positivity and the development of liver metastases was reported [49], confirming previous preclinical evidence [52].

We firstly evaluated HER-2 IHC score 0/1+ (negative) *vs.* IHC score 2+ with FISH gene amplification or 3+ (positive). However, none of the survival differences resulted in statistical significance. This may be due to the small number of HER-2 positive CRC patients (*n* = 6). Then, hypothesising that the lack of IHC expression of HER-2 may correlate with better outcomes, we exploratory compared HER-2 IHC score 0 to HER-2 IHC score 1+/2+/3+ survival data with a speculative purpose. Median survival measures (DFS, BPFS, ΔBPFS-DFS) were all longer for HER-2 score 0 CRC patients compared to those with HER-2 1+/2+/3+ tumors, but none of the comparisons reached statistical significance. Median OS reached borderline significance (30 months for HER-2 score 0 *vs.* 16 months for HER-2 1+/2+/3+, *p* = 0.078). Median postNCH-OS was significantly greater for HER-2 score 0 *vs.* HER-2 1+/2+/3+ CRC cancers (6.5 *vs.* 4.5 months, *p* = 0.01). Taken as a whole, the data suggest a worse prognosis in CRC tumors harboring HER-2 overexpression or amplification.

We recognize that our study presents several limitations. First of all, the cohort of patients is limited and highly selected. The occurrence of BM in CRC patients is uncommon, and usually restricted to long-term survivors. Secondly, the retrospective nature of the trial hampers the generalization of the results. This also considering that some information regarding medical or pathological charts was unavailable. Finally, although we recognize that clinical benefits are greatest in patients with HER-2 gene amplification or tumors strongly overexpressing HER-2 (graded 3+ by IHC), the small sample size of these categories in our series (*n* = 6) may have reduced the statistical power to demonstrate a survival difference. FISH analyses on HER-2 IHC score 1+ cases might have helped interpreting our data and explaining the slight difference in survival. However, since FISH is an expensive and time-consuming technique with potentially equivocal results [43] so we decided not to perform it. HER-2 expression revealed, in few cases, a focal staining within the tumor, suggesting the presence of subclonal HER-2 positive populations, as previously observed in gastric cancer [53]. Macroscopic sampling of primary tumors and selection of paraffin blocks for HER-2 testing, related to intratumoral heterogeneity, may result in possible false negative results.

A number of anti-HER-2 treatments (trastuzumab, lapatinib, TDM-1 and pertuzumab) have been shown to be effective for metastatic HER-2 positive breast or gastric cancers. Patients with BM from HER-2 positive cancers may also benefit from anti-HER-2 treatments, even if the limited penetration through the blood-brain barrier is the most likely explanation for lack of drug efficacy for BM. Since trastuzumab levels in cerebrospinal fluid were reported to be low when administered intravenously [54], intrathecal trastuzumab administration was tested and showed encouraging results against HER-2 positive leptomeningeal metastasis [55]. Lapatinib, an oral reversible tyrosine kinase inhibitor that blocks both ErbB1 and ErbB2, crossed the blood-brain barrier, and combined with capecitabine resulted in partial and complete responses of BM from HER-2 positive breast cancers [56].

There is cumulative evidence that HER-2 is involved in CRC cell growth and progression. The inhibition of HER-2 may cause a stop in HCA-7 colorectal cancer cell lines growth [57]. In preclinical models, lapatinib showed greater growth inhibition of TGFβ-activated colon cancer cells than antagonists targeting only ErbB1 or ErbB2 receptors [58]. Moreover, HER-2 amplification seems related to cetuximab resistance [36,59], suggesting that testing HER-2 inhibitors in HER-2 positive CRC population has a strong biological rationale. Actually, the combination of trastuzumab with either lapatinib or pertuzumab is being tested in a pioneer phase II study enrolling KRAS wild-type CRC patients harboring amplification of the HER-2 oncogene that have run out of options [60].

## 4. Materials and Methods

### 4.1. Patients and Tissue

We studied a cohort of 50 consecutive CRC patients who underwent neurosurgical resection of their BM among a series of nearly 500 cancer patients with BM treated, from 1999 until 2012, at the Neurosurgery Department of the University and General Hospital of Udine, Italy. Patients were eligible if they were 18 years or older at the time of diagnosis, had a pathologically confirmed diagnosis of CRC, and if they underwent neurosurgical treatment, either with radical or palliative intent. None of the patients were treated with trastuzumab-based regimens or with any other anti-HER-2 agent.

**Figure 3:**
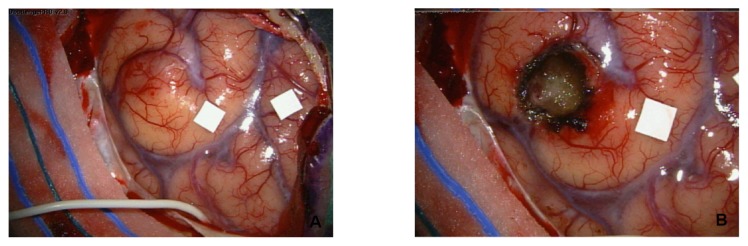
Macroscopic aspect of brain metastases from colorectal cancer before (**A**) and after (**B**) neurosurgical intervention.

In this study cohort, clinical data, including age, gender, Karnofsky PS, site of primary tumor, stage at diagnosis, number and location of BM, presence of extra-cranial disease, specific previous and following treatments, were retrospectively collected through revision of electronic medical records. The neurosurgical approach varied depending on the site and number of the secondary lesions, their size, and the risk of permanent neurological damages. Computer image guidance, visualized simultaneously, in all three orthogonal planes, allowed the surgeon to plan a tailored skin incision with minimal shave, and to identify the optimal trajectory for the shortest non-eloquent cortical route to target. In case of deep-seated lesions, affecting or even proximal to motor areas, intraoperative cortical mapping combined with preoperative functional MRI was carried out; awake craniotomy with brain mapping and neurophysiological monitoring of subcortical tracts were performed whenever indicated. Simple craniotomy, instead, was preferred for superficial, easily resectable metastases. Finally, multiple lesions were resected with a single surgical access (or with multiple approaches in the same surgical session) by using image guidance, which helped in localizing, even deep-seated, metastases distant from the cortical surface (Figure 3A,B). In selected cases with multiple distant lesions, a combined surgical and radiosurgical approach was adopted.

### 4.2. Tissue and Immunohistochemistry (IHC)

Formalin-fixed, paraffin-embedded samples were retrieved from the Pathology Department of the University and General Hospital of Udine, Italy, and compared with corresponding primary tumors in 37 out of 50 cases. Haematoxylin-Eosin (HE) slides of all cases were independently reviewed by a junior and a senior Pathologist in order to confirm the diagnosis and to choose the best inclusion for HER-2 assessment. Samples with representative neoplastic areas and minimum amount of necrosis or hemorrhagic component, in absence of surgical artifacts, were selected. HER-2 status was assessed on 4-μm sections by HerceptTest™ (Dako, Denmark) on an automatic platform (Dako Immunostainer Link48) according to manufactures instructions. A control slide consisting of three pelleted, formalin-fixed, paraffin-embedded human breast cell lines with staining intensity scores of 0, 1+, and 3+ (supplied with the HercepTest™ kit) was included in each staining run. In score 2+ cases HER-2 gene copy number has been analyzed by FISH (PathVysion HER-2 DNA Probe Kit Vysis, Wiesbaden, Germany) according to the manufacturer’s protocol. HER-2 gene amplification was defined as a HER-2: chromosome 17 ratio higher than 2. Analysis and photography were performed using a fluorescence microscope (Zeiss, Jena, Germany) equipped with a triple band pass filter for simultaneous detection of Spectrum Green, Spectrum Orange, and 4,6-diamidino-2 phenylindol dihydrochloride. HER-2 scoring was performed according to gastric cancer HER-2 status guidelines [53,61]. According to the literature, a case was considered positive for HER-2 status when revealed a 3+ IHC pattern or a 2+ IHC scoring with a concomitant gene amplification. With a speculative purpose, we also compared the outcomes of those cases with HER-2 IHC score higher than zero to those with no IHC expression. In 37 cases primary CRC was available and HER-2 expression of the resected brain lesion was compared to that of matched primary tumors.

### 4.3. Statistical Analysis

Data was summarized using standard descriptive statistics and frequency tabulations.

We defined disease-free survival (DFS) as the time between the date of initial diagnosis and date of stage IV diagnosis; brain progression-free survival (BPFS) as the time between the date of stage IV diagnosis and the date of surgery for BM; the time interval between the dates of BPFS and DFS was defined as ΔBPFS-DFS; overall survival after neurosurgery (postNCH-OS) as the time between the date of surgery for BM and the date of death, and OS as the time between the date of diagnosis and the death date.

Kaplan-Meier survival curves, with 95% Hall-Wellner Confidence Bands, were obtained and compared through the log rank test. A two sided *p* value of <0.05 was considered to be statistically significant. Concordance between IHC status on primary *vs.* metastatic sites was calculated as the ratio of concordant cases to total cases. All patients signed informed consent for the treatment they had received. Because this study was based on a retrospective analysis of the medical records, patient consent was not requested. However, the study was discussed and approved within the Investigational Review Board, to ensure adherence to institutional research policies and procedures related to human subjects. Also, an informative note was sent to the ethical committee of the University and General Hospital of Udine, Italy.

## 5. Conclusions

This is the first study reporting on HER-2 status of CRC brain metastases. The rate of HER-2 positive brain lesions was, overall, low but still reached 12%. The positivity of HER-2 on BM seems to be related to a worse prognosis, although further analysis on larger populations with pre-specified methodological criteria is warranted. The high concordance of HER-2 pattern between primary CRC tumors and corresponding BM might raises new hypotheses on the opportunity to test anti-HER-2 agents in this disease.

## Figures and Tables

**Table 1:** Demographics and clinical characteristics of the enrolled patients (*n* = 50).

	*N*	%
**Age, median & range**	65 (35–82)	

**Gender**		
Female	20	40
Male	30	60

**PS (Karnofsky)**		
<70	21	42
≥70	18	36
N.A	11	22

**Primary Tumor**		
Colon	19	38
Rectum	17	34
N.A	14	28
**T**		
Resected	37	74
Still present	10	20
N.A	3	6

**Stage at diagnosis**		
I	1	2
II	3	6
III	19	38
IV	19	38
N.A	8	16

**N^∘^of SNC lesions**		
1	37	74
2–4	9	18
>4	3	6
N.A	1	2

**BM at diagnosis**		
Yes	5	10
No	45	90

**Site of BM**		
Supratentorial	32	64
Subtentorial	17	34
Both	1	2

**Extra CNS lesions**		
Lung	31	62 *
Liver	26	52 *
Other	18	36 *

N.A = not available;

* = the rate is calculated on the overall population (*i.e.*, the sum is not 100%).

**Table 2:** Pre-neurosurgical treatments.

	*N*	%
**Surgery**	37	74

**Radiotherapy**	3	6

**Chemotherapy**		
Adjuvant		
Yes	20	40
No	24	48
N.A	6	12
Palliative		
Yes	29	58
No	9	18
N.A	12	24

**Chemotherapy**		
5-Fluorouracil	30	60
Oxaliplatin	16	32
Irinotecan	16	32

**Biological agents**		
Bevacizumab	0	0
Cetuximab	4	8
Panitumumab	0	0

**Table 3:** Post-neurosurgical treatments.

	*N*	%
**Gamma-knife**		
Yes	5	10
No	36	72
N.A	9	18

**WBRT**		
Yes	21	42
No	29	58

N.A = not available; WBRT = Whole Brain Radiotherapy.

**Table 4:** Colorectal (*n* = 37) and brain metastases (*n* = 50) HER-2 expression and immunohistochemical score.

HER-2	Colorectal tumor (*n* = 37)		Brain metastases (*n* = 50)	

	N	%	N	%
**0**	31	83.8	36	72
**1+**	3	8.1	8	16
**2+**	2*	5.4 *	2 *	4 *
**3+**	1	2.7	4	8
**N.A**	13	-	0	0

N.A = not available;

* = all FISH amplificated.

**Table 5:** IHC HER-2 status concordance between primary tumors and corresponding brain metastases in 37 matched cases *.

HER-2		BM		Total

		0 or 1+ *N* (%)	2+ or 3+ *N* (%)	
**CRC**	**0 or 1+ N (%)**	31 (83.7)	3 (8.1)	34 (91.8)
	**2+ or 3+ N (%)**	1 (2.7)	2 (5.4)	3 (8.1)

	**tot**	32 (86.4)	5 (13.5)	37

* All IHC score 2+ cases presented HER-2 gene amplification at FISH.
